# Bilirubin and risk of ischemic heart disease in Korea: a two-sample Mendelian randomization study

**DOI:** 10.4178/epih.e2019034

**Published:** 2019-07-12

**Authors:** Christina Jeon, Ji-Young Lee, Sun Ju Lee, Keum Ji Jung, Heejin Kimm, Sun Ha Jee

**Affiliations:** 1 Department of Epidemiology and Health Promotion, Institute for Health Promotion, Graduate School of Public Health, Yonsei University, Seoul, Korea; 2 Department of Public Health, Graduate School, Yonsei University, Seoul, Korea

**Keywords:** Bilirubin, Myocardial ischemia, Two-sample Mendelian randomization study, Korea

## Abstract

**OBJECTIVES:**

Bilirubin is an endogenous antioxidant that protects cells against oxidative stress. Increased plasma levels of bilirubin have been associated with a reduced risk of ischemic heart disease (IHD) in previous studies. Nonetheless, whether those associations reflect a true protective effect of bilirubin on IHD, rather than confounding or reverse causation, remains unknown. Therefore, we applied two-sample Mendelian randomization to evaluate the causal association between bilirubin levels and IHD risk in a Korean population.

**METHODS:**

A total of 5 genetic variants—*TRPM8* (rs10490012), **USP40** (rs12993249), *ATG16L1* (rs2119503), *SLCO1B1* (rs4149014), and *SLCO1B3* (rs73233620)—were selected as genetic instruments for serum bilirubin levels using a communitybased cohort, the Korean Genome and Epidemiology Study, comprising 33,598 subjects. We then evaluated their impact on IHD using the Korean Cancer Prevention Study-II cohort.

**RESULTS:**

Among the 5 instrumental variables that showed significant associations with serum bilirubin levels, rs12993249 (**USP40**) showed the most significant association (p<2.36×10^-105^). However, we found no significant association between serum bilirubin levels and IHD. Sensitivity analyses demonstrated a consistent association, suggesting that our observations were robust.

**CONCLUSIONS:**

Using two-sample Mendelian randomization, we found no association between serum bilirubin levels and IHD. Further studies that confirm the observed interactions among other ethnicities are warranted.

## INTRODUCTION

Bilirubin is an endogenous antioxidant that protects cells against oxidative stress [1]. As heme oxygenase-1 facilitates oxidation to generate biliverdin and other substances, it produces anti-inflammatory and antioxidative products. Due to the cytoprotective effect of bilirubin, elevated activity of oxygenase or a rate-limiting enzyme in heme, which can be caused by genetic or environmental factors, has been reported to be beneficial for health overall and for injuries [2].

Ischemic heart disease (IHD) remains a major health issue worldwide. Previous evidence has shown that inflammation plays a key role in the initiation and progression of cardiovascular disease [3]. In addition, in previous studies, increased plasma levels of bilirubin have been associated with a reduced risk of IHD [4,5]. Nonetheless, whether those associations reflect a true protective effect of bilirubin on IHD, rather than confounding or reverse causation, remains unknown [6]. Furthermore, some of those studies were conducted in populations at high risk. A recent meta-analysis of 11 studies and a Mendelian randomization (MR)study reported no relationship between bilirubin levels and IHD [7]. To date, the relationship between bilirubin levels and IHD is not well-characterized.

Therefore, in the present study, we sought to investigate whether bilirubin levels are causally related to IHD risk using the two-sample MR approach in a Korean population.

## MATERIALS AND METHODS

### Data sources for the study exposure

Genetic associations with bilirubin levels were obtained from the Korean Genome and Epidemiology Study (KoGES). The KoGES, which is categorized into population-based and gene-environment model studies, is a consortium project consisting of 6 prospective cohort studies supported by the government funding. Over 223,000 participants have been recruited, of whom 35,000 participants from population-based studies (KoGES_Ansan and Ansung Study and KoGES_HEXA Study) and who had epidemiological information and DNA samples were selected. The characteristic details of the KoGES have been previously described [8].

In total, 1,402 of the 35,000 participants were excluded through the quality control procedure (sex inconsistency: n=127; <97% call rate or excessive heterozygosity: n=635; excessive singletons: n=69; cryptic first-degree relatives: n=393; and withdrawals and blind duplicates: n=178). Finally, a total of 33,598 participants were selected for further analysis (Supplementary Material 1). Genomic DNA was extracted from peripheral blood monocytes by standard procedures, and single-nucleotide polymorphism (SNP) data was obtained using Korean Chip (K-CHIP) as part of the K-CHIP consortium. K-CHIP, which contains 830,000 SNPs customized for the Korean population, was designed by the Center for Genome Science of the Korea National Institute of Health (http://nih.go.kr/menu.es?mid=a50303010100). To increase the coverage of the genome-wide association study (GWAS), we conducted imputation analysis using the 1000 Genomes (1000G) Phase 3 dataset of East Asian populations (Japanese in Tokyo [JPT], Chinese in Beijing [CHB]) as a reference panel. Haplotype phasing with sample genotypes from the 1000G reference panel was performed using IMPUTE2 software. For quality control, SNPs were excluded prior to the analysis using the following criteria: (1) deviation from Hardy-Weinberg equilibrium (HWE) with a p-value less than 10^-4^ and (2) minor allele frequency (MAF) less than 1%.

### Genetic associations of variants with exposure and selection of the genetic instrumental variables

Linear regression with adjustment for age and sex was performed to examine the associations of genetic variants with bilirubin. Statistical analyses were performed using PLINK version 1.07. We obtained 1,784 SNPs with a genome-wide significance level (p<10^-8^). For standard two-sample MR, it is crucial to confirm that the instruments for the exposure are strongly independent. Thus, we examined the clumping test to estimate the linkage disequilibrium between selected SNPs using 1000G data (https://mrcieu.github.io/TwoSampleMR). Finally, a total of 6 independent SNPs (rs10490012, rs12993249, rs2119503, rs4149014, rs2219067, and rs73233620) were selected as instrumental variables for serum bilirubin levels (r^2^>0.001). Among them, we removed 1 palindromic SNP (rs2219067) from further analysis.

### Data sources for the study outcome

IHD was defined as our outcome. The outcome data were obtained from the Korean Cancer Prevention Study-II (KCPS-II) cohort, which comprises 156,701 participants (94,840 men and 61,861 women) who received a medical examination at 18 health promotion centers in Seoul and Gyeonggi Province, Korea between 2004 and 2013. Regular or obligatory health check-ups and health issues were most common reasons for their visits. In addition, participants received regular follow-up. Details about the KCPS-II cohort can be found in a previously published cohort profile article [9]. Among the participants, subjects with genotype and phenotype data who provided written consent were selected for further analysis. GWAS analysis was conducted using a logistic regression model with adjustment for age and sex (MAF≥0.01, p for HWE test≥0.0001). Odds for IHD risk were used when calculating ratio estimates.

### Associations of genetic variants with the outcome

A total of 1,910 cases and 11,945 randomly selected controls were used to examine the associations of genetic variants with IHD. Genotyping was carried out with K-CHIP, using the same method as described above. After genotyping, we also performed imputation analysis using the 1000G Phase 3 dataset for East Asian populations (JPT, CHB) as a reference panel to infer missing genotypes. The quality control procedures for SNPs were conducted using the same procedure as for the selection of the study exposure: (1) deviation from HWE (p<10^-4^) and (2) MAF<1%. Logistic regression adjusted for age and sex was performed to determine genetic associations with IHD. Statistical analyses were performed using PLINK version 1.07 (http://zzz.bwh.harvard.edu/plink/).

### Mendelian randomization

**Figure 1.**
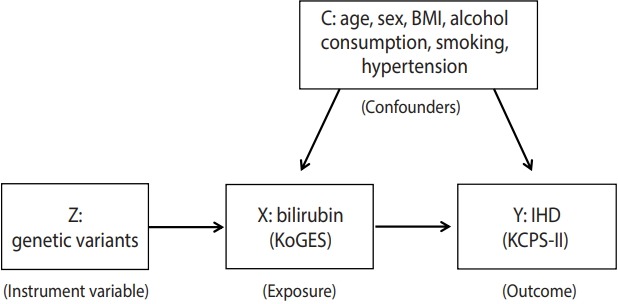
Overview of the study design. BMI, body mass index; KoGES, Korean Genome and Epidemiology Study; KCPS-II, Korean Cancer Prevention Study-II; IHD, ischemic heart disease.

Two-sample MR analyses were performed to investigate the existence of a causal relationship between bilirubin and IHD (Figure 1). Two-sample MR is an approach used to investigate potential causal relationships between modifiable risk factors and an outcome of interest, using genetic variants as an instrumental variable (IV). MR studies are more reliable than observational studies, and they provide proper guidelines for interventional research and public health interventions for population genetics [10]. Unlike single-sample MR, two-sample MR uses summary association results from non-overlapping sets of individuals obtained from the published literature or results estimated directly from individual-level participant data. The key assumptions of two-sample MR are (1) the IV is causally related to the risk factor; (2) confounding factors of the association between risk factors and the outcome should not be related to the IV; and (3) the IV only affects the outcome though its effect on risk factors [10,11].

The odds of IHD risk were divided by the β coefficient of bilirubin levels to determine ratio estimates for each IV. The effects of the individual genetic instruments were combined using inverse-variance weighted (IVW) analysis, resulting in a weighted mean estimate of the risk for IHD per 1-standard deviation increase in serum bilirubin levels. As the IVW method assumes that all genetic variants satisfy the IV assumptions, sensitivity analyses were performed with MR–Egger regression and the weighted median estimation method. A leave-one-out sensitivity analysis, leaving outlying variants out of the MR analysis, was performed, as weak instruments were used to predict serum bilirubin levels (Supplementary Material 2). A radial plot was applied to assess heterogeneity across genetic variants and to detect pleiotropic variants (Supplementary Material 3).

The MR package in R statistical software (MR Base website: https://github.com/MRCIEU/TwoSampleMR) was used to perform the two-sample MR analysis.

### Ethics statement

The Severance Medical Ethics Committee approved this study (no. 4-2011-0277).

## RESULTS

Table 1 shows the associations of the 5 SNPs (rs10490012, rs12993249, rs2119503, rs4149014, and rs73233620) selected as genetic instruments for serum bilirubin levels with IHD risk. For the genetic association analysis of bilirubin, 5 SNPs showed sufficient strength to minimize the effects of the weak instrument bias in our analysis of bilirubin levels (F-statistics ranged from 37.61 to 450.09). Among the 5 SNPs, rs12993249 (located near the **USP40** gene on chromosome 2) showed the most significant relationship with bilirubin levels (β [standard error, SE]=0.060 [0.003]; p=2.36 ×10^-105^), followed by rs73233620, which is located on the *SLCO1B3* gene (β [SE]=0.057 [0.003]; p=1.44×10^-65^) and was previously identified in a GWAS analysis of bilirubin. No significant genetic associations with IHD risk were found (Table 1).

**Figure 2.**
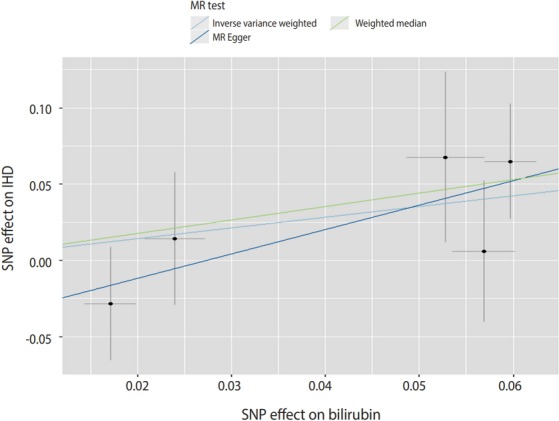
MR analysis regarding the effect of serum bilirubin levels on IHD risk using the genetic instruments. Scatter plot and causal effect of serum bilirubin on IHD risk. The X-axis presents the effect of the genetic instrument of serum bilirubin concentration (in mg/dL); the Y-axis presents the causal effect of the genetic instrument on IHD risk. MR, Mendelian randomization; IHD, ischemic heart disease; SNP, single-nucleotide polymorphism.

Table 2 shows the results of the two-sample MR analysis for serum bilirubin levels on IHD risk. Using the 5 genetic variants as IVs, we found that bilirubin levels had no significant effect on the risk of IHD in East Asians (IVW: OR, 2.03; 95% confidence interval [CI], 0.87 to 4.70; p=0.100) (Table 2 and Figure 2). The weighted median analyses showed that there was also no significant association between bilirubin levels and risk of IHD (OR, 2.41; 95% CI, 0.89 to 6.57; p=0.085) (Table 2 and Figure 2). MR-Egger regression showed non-significant, but much higher risk (OR, 4.94; 95% CI, 0.68 to 35.91; p=0.213) compared to the estimates obtained using the IWV and weighted median MR methods. Because the intercept from the MR-Egger regression analysis showed no significant deviation from zero, no evidence for directional pleiotropy was observed. A radial plot showed that no significant outliers existed among the 5 SNPs (Supplementary Material 3).

## DISCUSSION

We conducted a two-sample MR study and found that 5 genetic variants were significantly associated with bilirubin levels. Although our results do not provide evidence for a positive effect of bilirubin levels on IHD risk, to our knowledge, this is the first two-sample MR study to examine the effect of bilirubin levels on IHD risk among an East Asian population.

Previous epidemiological studies have shown contradictory results for associations between bilirubin levels and IHD risk. A recent meta-analysis found an inverse association between total bilirubin levels and the risk of cardiovascular disease, which was independent of established risk factors [4]. However, other studies indicated that higher levels of bilirubin showed direct or null associations with cardiovascular disease risk [12,13]. Our non-causal findings are in line with recent MR studies among European populations on IHD that used *UGT1A1* and *SLCO1B1*, which are known to be bilirubin metabolism genes, and suggested that bilirubin is not causally associated with IHD risk [7] (Supplementary Material 4). Similarly, a previous MR study on bilirubin levels in a Korean population conducted by our research team demonstrated a non-causal association between SNPs at the UGT1 locus and bilirubin levels [14]. In this context, our study has several strengths, including its design and methods. First, we selected variants of *SLCO1B1*/3 as genetic instruments derived from an analysis of GWAS data from a Korean population. Because the allele frequencies of *SLCO1B1*/3 are relatively low or monomorphic in European populations, GWAS of bilirubin based on European ancestry could not reveal significant novel findings in or nearby this gene, in contrast to the findings of Korean GWAS for *SLCO1B3* variants [15]. Therefore, the genetic instruments used in our study may be applicable to East Asian genetic diversity datasets. Second, unlike previous MR studies that used summary data from large consortia, our study used a single dataset without a population substructure, thereby avoiding potential heterogeneity. Third, two-sample MR studies are unlikely to have any bias that underestimates true causal effects as a result of the weak instrument [16-18].

Nonetheless, our study has several potential limitations. First, the 5 selected instruments (SNPs) may not be applicable or useful in other ethnic groups. Therefore, our study findings may not be generalized to other cohorts with different ethnicities. Regarding the *SLCO1B1*/3 (rs4149014, rs73233620) variants, a comparison of 1000G Phase 3 data for Caucasian (CEU) and East Asian populations revealed notable distinctions in the MAF (CEU: 3.0% for rs4149014 and 5.0% for rs73233620; East Asian: 33.3% for rs4149014 and 17.8% for rs73233620) (Supplementary Material 5). Alternative instruments may need to be discovered in CEU populations. In addition, although MR is suggested to be a fruitful method capable of overcoming some of the limitations of observational studies, the current findings cannot exclude potential inherent biases and limited causal inferences [19].

Nevertheless, identifying additional loci associated with bilirubin would yield loci with smaller effect sizes and weights in the combined analyses.

In conclusion, our study provides evidence that bilirubin levels may not be causally related to the risk of IHD. While no association between serum bilirubin levels and IHD was found, further studies that replicate the observed interactions among other ethnicities are warranted.

## Figures and Tables

**Table 1.** Associations of individual genetic instruments for serum bilirubin levels with IHD risk

SNP	Nearby gene	Chromosome	Effect allele	Bilirubin		IHD	
				β (SE)	p-value	OR (95% CI)	p-value
rs10490012	*TRPM8*	2	T	-0.017 (0.003)	1.15×10^-10^	1.03 (0.96, 1.11)	0.434
rs12993249	*USP40*	2	G	0.060 (0.003)	2.36×10^-105^	1.07 (0.99, 1.15)	0.086
rs2119503	*ATG16L1*	2	A	0.053 (0.004)	4.63×10^-37^	1.07 (0.96, 1.19)	0.226
rs4149014	*SLCO1B1*	12	G	-0.024 (0.003)	3.72×10^-14^	0.99 (0.91, 1.07)	0.744
rs73233620	*SLCO1B3*	12	G	0.057 (0.003)	1.44×10^-65^	1.01 (0.92, 1.10)	0.897

IHD, ischemic heart disease; SNP, single-nucleotide polymorphism; SE, standard error; OR, odds ratio; CI, confidence interval.

**Table 2.** MR for the effects of serum bilirubin on ischemic heart disease risk

Variables	OR (95% CI)	p-value	No. of SNPs
Inverse-variance weighted	2.03 (0.87, 4.70)	0.100	5
Weighted median	2.41 (0.89, 6.57)	0.085	5
MR-Egger regression			5
Estimate	4.94 (0.68, 35.91)	0.213	5
Intercept, β (SE)	-0.04 (0.05)	0.403	5

MR, Mendelian randomization; OR, odds ratio; CI, confidence interval; SNP, single-nucleotide polymorphism; SE, standard error.
