# Supplementary material for: Bilirubin and risk of ischemic heart disease in Korea: a two-sample Mendelian randomization study
Source: Epidemiol Health. 2019 Jul 12;41:e2019034. doi: 10.4178/epih.e2019034 (PMC6759489; doi:10.4178/epih.e2019034)
Supplement: Supplementary file 1 [file epih-41-e2019034-supplementary.pdf]

Supplementary Material 1. Flow chart of study population in two-sample Mendelian Randomization

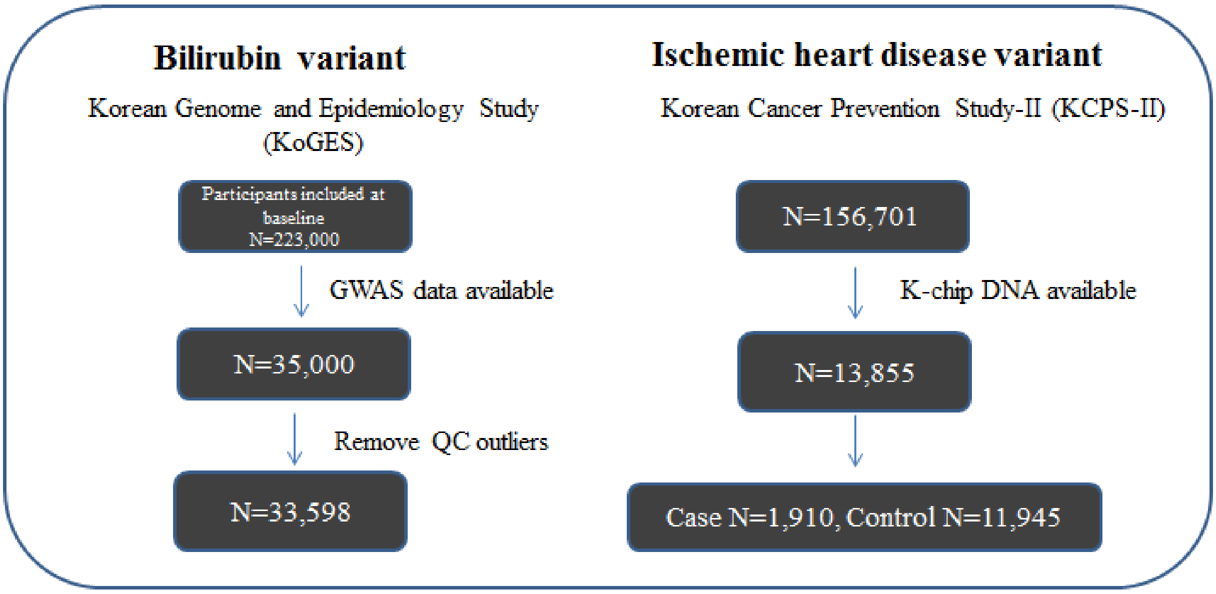


*KNIH: Korea National Institute of Health

Supplementary Material 2. Leave-one-out analysis

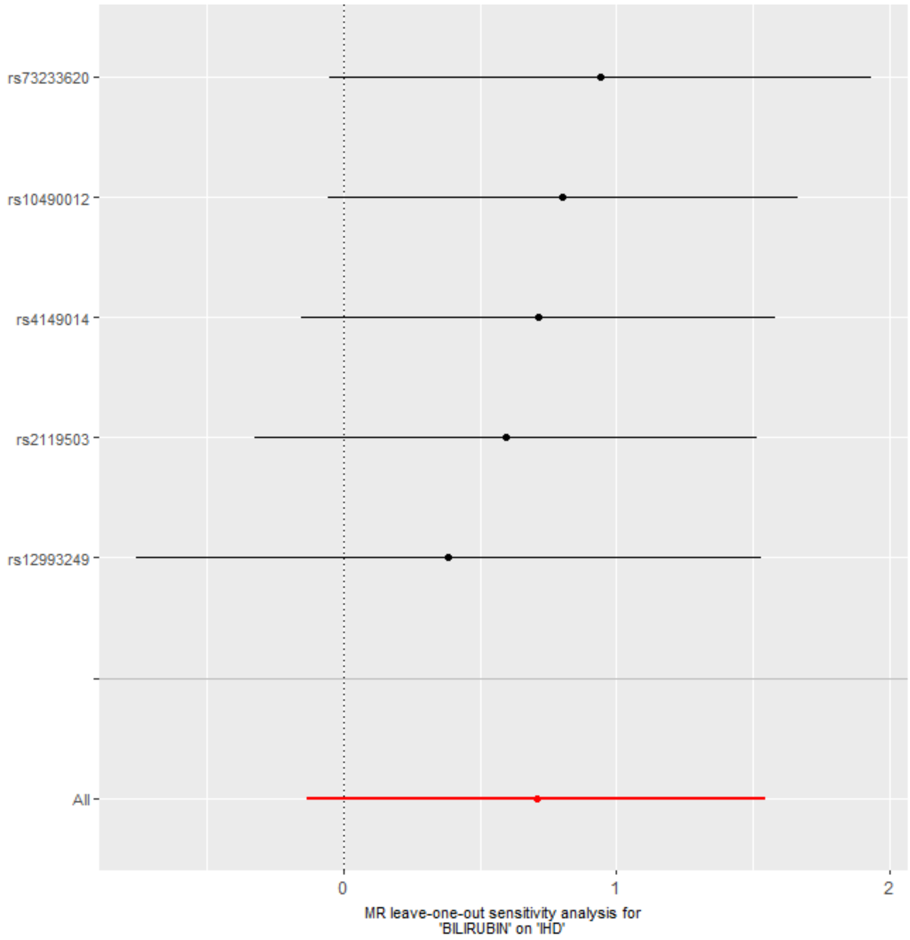

Supplementary Material 3. Radial plot showing no significant outliers

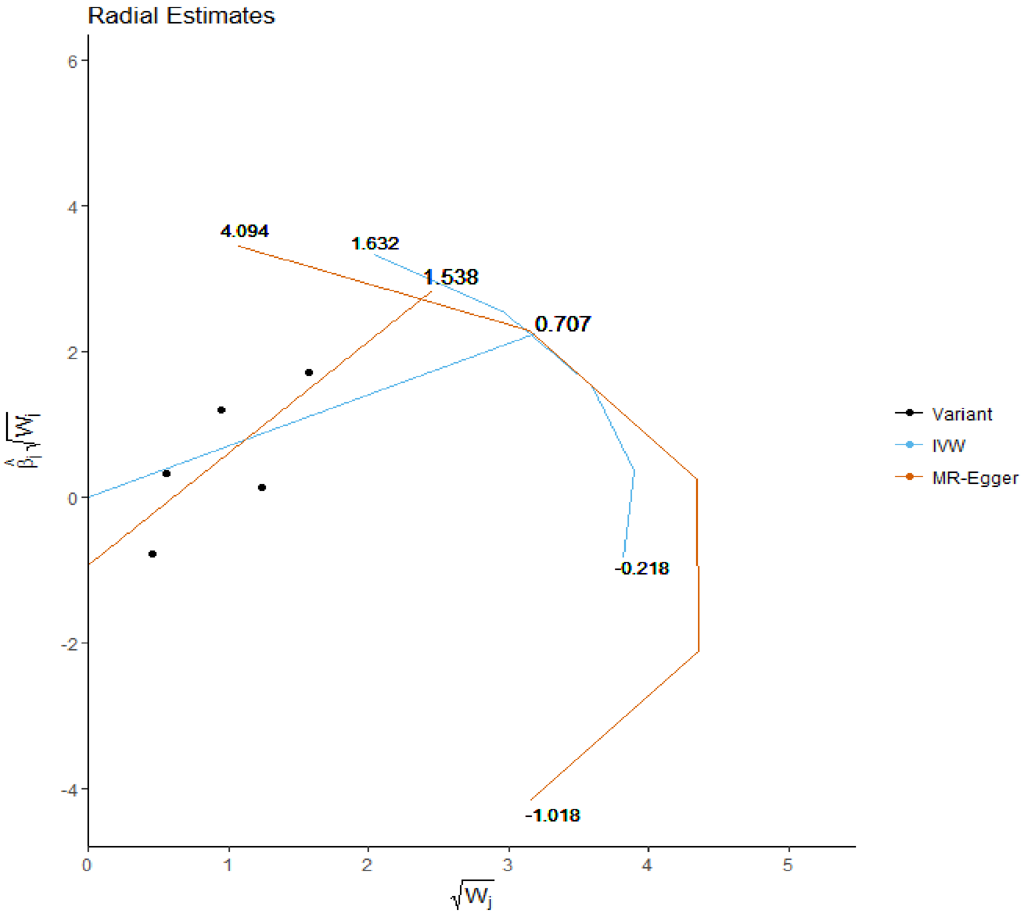

Supplementary Material 4. Association of ischemic heart disease in bilirubin genetic variants based on two-sample Mendelian randomization using SNPs from previously identified from European ancestry

| | | | | Reported GWAS Studies | | Current study on 2-sample Mendelian randomization analysis (IVW) | |
|---|---|---|---|---|---|---|---|
| SNP | Genes | Chr | MAF | *p*-value | Reference | Beta (*SE*) | *p*-value |
| rs6742078 | *UGT1A1* | 2 | 0.32 | 5E-324 | (1) | | |
| rs887829 | *UGT1A1* | 2 | 0.32 | 5E-324 | (1),(2) | | |
| rs11891311 | *UGT1A1* | 2 | 0.35 | 1E-235 | (1),(2) | 0.49 (0.28) | 0.0809 |
| rs4149056 | *SLCO1B1* | 12 | 0.15 | 7E-13 | (1) | | |
| rs2117032 | *SLCO1B3* | 12 | 0.46 | 3E-14 | (2) | | |

(1) Johnson AD, Kavousi M, Smith AV et al: Genome-wide association meta-analysis for total serum bilirubin levels. Hum Mol Genet 2009; 18: 2700–2710.

(2) Sanna S, Busonero F, Maschio A et al: Common variants in the SLCO1B3 locus are associated with bilirubin levels and unconjugated hyperbilirubinemia. Hum Mol Genet 2009; 18: 2711–2718

Abbreviation, GWAS; genome-wide association study. Chr: chromosome, MAF; minor allele frequency, SE; standard error, IVW: inverse variance weighted

Supplementary Material 5. Ethnic differences in allele frequencies of *SLCO1B1* and *SLCO1B3* based on 1000 Genomes reference

| | **rs4149014** | | **rs73233620** | |
|---|---|---|---|---|
| Population | Ref Allele | Alt Allele | Ref Allele | Alt Allele |
| African | 0.940 | 0.060 | 0.775 | 0.225 |
| East Asian | 0.667 | 0.333 | 0.822 | 0.178 |
| Europe | 0.981 | 0.019 | 0.975 | 0.025 |
| South Asian | 0.96 | 0.04 | 0.97 | 0.03 |
| American | 0.97 | 0.03 | 0.95 | 0.05 |

Ref : reference, Alt : alternative
